# Thermodynamics of binding of divalent magnesium and manganese to uridine phosphates: implications for diabetes-related hypomagnesaemia and carbohydrate biocatalysis

**DOI:** 10.1186/1752-153X-2-15

**Published:** 2008-07-15

**Authors:** Corbin J Zea, Gulden Camci-Unal, Nicola L Pohl

**Affiliations:** 1 Department of Chemistry and the Plant Sciences Institute, Iowa State University, Gilman Hall, Ames, IA, 50011-3111, USA

## Abstract

**Background:**

Although the necessity of divalent magnesium and manganese for full activity of sugar nucleotidyltransferases and glycosyltransferases is well known, the role of these metal cations in binding the substrates (uridine 5'-triphosphate, glucose-1-phosphate, *N*-acetylglucosamine-1-phosphate, and uridine 5'-diphosphate glucose), products (uridine 5'-diphosphate glucose, uridine 5'-diphosphate *N*-acetylglucosamine, pyrophosphate, and uridine 5'-diphosphate), and/or enzyme is not clearly understood.

**Results:**

Using isothermal titration calorimetry we have studied the binding relationship between the divalent metals, magnesium and manganese, and uridine 5'-phosphates to determine the role these metals play in carbohydrate biosynthesis. It was determined from the isothermal titration calorimetry (ITC) data that Mg^+2 ^and Mn^+2 ^are most tightly bound to PP_*i*_, K_b _= 41,000 ± 2000 M^-1 ^and 28,000 ± 50,000 M^-1 ^respectively, and UTP, K_b _= 14,300 ± 700 M^-1 ^and 13,000 ± 2,000 M^-1 ^respectively.

**Conclusion:**

Our results indicate that the formal charge state of the phosphate containing substrates determine the binding strength. Divalent metal cations magnesium and manganese showed similar trends in binding to the sugar substrates. Enthalpy of binding values were all determined to be endothermic except for the PP_*i *_case. In addition, entropy of binding values were all found to be positive. From this data, we discuss the role of magnesium and manganese in both sugar nucleotidyltransferase and glycosyltransferase reactions, the differences in metal-bound substrates expected under normal physiological metal concentrations and those of hypomagnesaemia, and the implications for drug design.

## Background

The roles that nucleotides play in cellular metabolism range in scope from their use as enzyme substrates to regulators for numerous biochemical pathways. Because of their biological importance and the requirement of divalent metals for biological activity, the binding relationship between various metals and nucleotides has been well documented to shed light on the role of metal-nucleotide complexes in the conformational changes that occur in DNA biosynthesis [1-4] and the energy pathways that involve ATP [5-12].

Recent studies suggest that certain metabolic disorders are linked to serum magnesium concentrations in a way that can be partially explained by these relative metal-binding equilibria. For example, individuals with hypomagnesaemia – reduced serum magnesium concentration – have an increased incidence of metabolic disorders such as type 2 diabetes mellitus [13-20] and high blood pressure [13]. Simple oral administration of magnesium chloride can increase serum magnesium concentrations and decrease total cholesterol and HDL levels while increasing LDL and insulin sensitivity in these patients [21-23]. In patients with high cholesterol and hypomagnesaemia, magnesium treatment is attributed to increasing the effectiveness of statin pharmaceuticals by shifting the equilibrium to the formation of a Mg^+2^-ATP complex which is a necessary intermediate in the cholesterol biosynthetic pathway [24]. Surprisingly, searches for an explanation of the effect of magnesium supplementation on insulin levels so far have focused only on metal effects at the DNA level [1] and not on the effects of metals on the relative amounts of the substrates involved in glucose metabolism. Such discussions are hindered by the lack of reports of the magnesium binding affinities to these substrates.

**Scheme 1:**
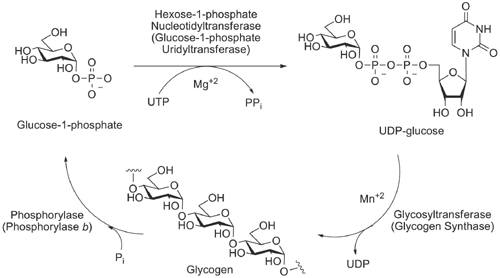
Biochemical pathway for the synthesis and degradation of glycogen. General classification and the specific names of biocatalysts associated with carbohydrate biosynthesis are shown.

The biochemical pathway that synthesizes glycogen and degrades it (Scheme 1) plays an important role in cellular function and metabolism. This pathway is responsible for the storage of glucose and glycogen as well as the regulation of blood glucose levels. For this reason glycogen synthase as well as phosphorylase *b *have been targets of diabetic drug targets [25-28]. It has also been shown that the biocatalyst that activates glucose as ADP-glucose in plants plays a regulatory role in starch biosynthesis [29-31] which tags all three biocatalysts as contributing to the regulation of glucose concentrations.

While it is widely known that divalent cations, in particular Mg^+2 ^and Mn^+2^, play a very important role in carbohydrate biocatalysis, the actual mechanism by which the metal interacts with the ligand and the enzymes is not clearly understood. This has important implications in drug development; whether the cation generally binds tightly to the protein active site or binds to the ligand before ligand binding to the protein is an important consideration in designing effective inhibitors of this interaction. It is well documented that uridine triphosphate (UTP), pyrophosphate (PP_*i*_), glucose-1-phosphate (Glc-1-P), and uridine diphosphate glucose (UDP-Glc) all bind and act as inhibitors [32-35], whereas uridine diphosphate (UDP) was demonstrated by Gillett and coworkers [36] to not bind to UDP-Glc pyrophosphorylase. This result is confusing since other researchers have determined UDP to be an inhibitor of UDP-Glc pyrophosphorylase [33,34]. This raises the question of how inhibitor binding is related to inhibitory affect. Herein, we discuss the binding of Mg^+2 ^and Mn^+2 ^with UTP, UDP, uridine monophosphate (UMP), UDP-Glc, and PP_*i *_in order to gain an understanding in how and why sugar nucleotidyltransferases and glycosyltransferases (type B) are metal dependant, and to determine in what order metal binding occurs (i.e. does the metal bind to the ligand or biocatalyst first) and what role that has on catalytic activity or inhibition.

## Results and discussion

**Figure 1:**
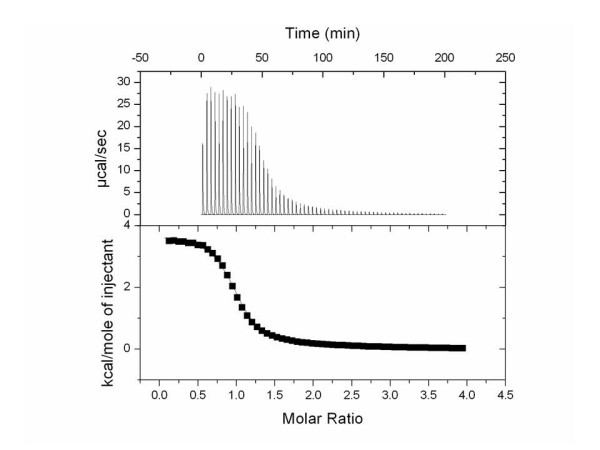
Thermogram (top) and binding isotherm (bottom) showing the addition of 75 mM MgCl_2 _(syringe) into 2.1 mM UTP (cell) in 100 mM HEPES at pH 7.6 and 37°C.

**Figure 2:**
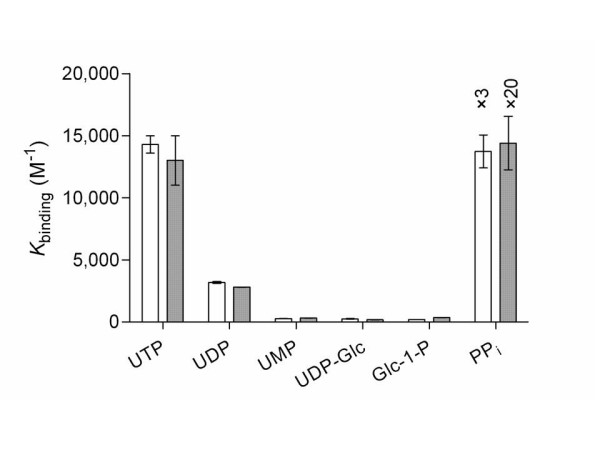
Binding constants of phosphates bound to Mg^+2 ^(white) and Mn^+2 ^(gray).

While the binding of Mg^+2 ^to nucleotide phosphates has been studied previously [5,7,11], the binding relationship between the metals, Mg^+2 ^and Mn^+2^, and uridine phosphates have not been described. Because the primary interaction between the metal and nucleotides occurs between the phosphates and the metal one would expect the binding parameters between ATP and UTP to be similar. It would also be expected that little binding would be lost by the addition of glucose to UDP to form UDP-glucose. To address these questions, we determined the thermodynamics of binding of Mg^+2 ^and Mn^+2 ^to UTP, UDP, UMP, UDP-Glc, Glc-1-P, Glc, U, and PP_*i *_at the physiologically relevant temperature of 37°C using isothermal titration calorimetry (ITC) by the direct titration of a solution of magnesium or manganese (II) chloride into a solution containing the phosphate containing compound being studied (Figure 1). It was determined from the ITC data that Mg^+2 ^and Mn^+2 ^are most tightly bound to PP_*i*_, K_b _= 41,000 ± 2000 M^-1 ^and 28,000 ± 50,000 M^-1 ^respectively, and UTP, K_b _= 14,300 ± 700 M^-1 ^and 13,000 ± 2,000 M^-1^respectively (Figure 2). The binding parameters determined for the binding of Mg^+2 ^and Mn^+2 ^to UDP and UMP were found to be less than that of UTP. The degree of association between Mg^+2 ^and Mn^+2 ^decreases as phosphates are removed (UTP > UDP > UMP). However, as expected, the binding parameters measured were comparable to previously reported results for the binding of Mg^+2 ^to ATP, ADP, AMP, and UMP [1,5-7]. In addition, as expected, there was no measurable binding of Mg^+2 ^and Mn^+2 ^to glucose and uridine which do not contain negatively charged phosphates.

Comparison of the binding constants for the binding of Mg^+2 ^and Mn^+2 ^to UDP-Glc to the binding of Mg^+2 ^and Mn^+2 ^to UDP affords an approximately 10-fold decrease in binding. This significant decrease in binding due to the addition of glucose was not expected; UDP-Glc is often drawn directly bound to these divalent metals. In contrast, the binding values associated with the binding of Mg^+2 ^and Mn^+2 ^to Glc-1-P were found to be similar in strength to the binding of Mg^+2 ^and Mn^+2 ^to UMP. This data suggests that the binding of Mg^+2 ^and Mn^+2 ^to phosphates is dependent on the binding environment of the phosphates involved in the coordination of the metals, Mg^+2 ^and Mn^+2^. For comparison, binding affinities to *N*-acetylglucosamine, *N*-acetylglucosamine-1-phosphate, and uridine 5'-diphosphate *N*-acetylglucosamine were also measured. From the binding constants the following pattern of affinities emerges: PP_*i *_> UTP > UDP > UMP ≈ UDP-Glc ≈ Glc-1-P ≈ GlcNAc-1-P > UDP-GlcNAc. This data demonstrates a great effect on metal affinities upon substitution at phosphate. If the phosphate has no substituents covalently bound, as in the case of PP_*i*_, the binding constant is large. However, in the case of UMP, UDP-Glc, and Glc-1-P, in which the substrate is bound directly to the binding phosphate, the binding constant is significantly smaller. Finally, in the examples of substrates not having phosphate groups present, GlcNAc, Glc and U, the binding of Mg^+2 ^and Mn^+2 ^to the substrates was so weak as to preclude accurate binding affinity determinations, which indicates the importance that the charged phosphates play in binding divalent metals.

Thermodynamic values for the titrations were determined and for all cases, except for the titration of pyrophosphate by Mg^+2 ^and Mn^+2^, the binding enthalpies were endothermic. All titrations afforded a positive change in entropy, which correlates to an increase in disorder caused by the loss of a water shell around the metal and phosphate compound as the metal coordinates to the phosphate compound. This increase in disorder allowed the overall coordination of Mg^+2 ^and Mn^+2 ^to the phosphate to be thermodynamically allowed with the free energy following a similar pattern as the binding values (PP_*i *_> UTP > UDP > UMP ≈ UDP-Glc ≈ Glc-1-P ≈ GlcNAc-1-P > UDP-GlcNAc).

**Figure 3:**
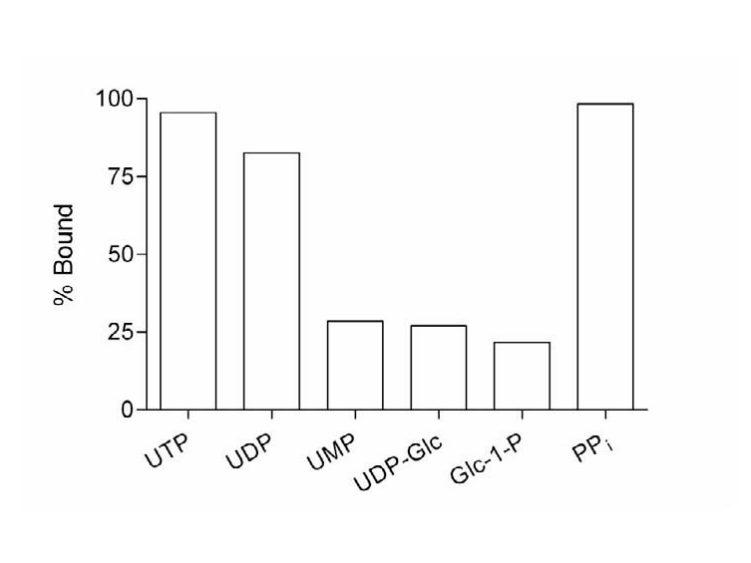
Percent of phosphate bound to Mg^+2 ^(white) at the intracellular concentration of 1.5 mM. No measurable binding is observed for Mn^+2^.

Although Mg^+2 ^and Mn^+2 ^bind at very similar strengths to phosphates, the intracellular concentrations of Mg^+2 ^and Mn^+2 ^vary greatly. Although metal concentrations can vary depending on cell state, type, and organelle, given intracellular concentrations of Mg^+2 ^and Mn^+2 ^of 1.5 nM [37] and 10 nM [38] respectively, the amount of phosphate expected to be bound to Mg^+2 ^and Mn^+2 ^varies greatly as shown in Figure 3. At these physiological metal concentrations, the concentration of Mn^+2 ^is not large enough to shift the equilibrium of binding in the direction of metal-phosphate complex formation. However, all of the phosphates show a measurable degree of binding to Mg^+2 ^at intracellular concentrations of Mg^+2^. Uridine triphosphate and PP_*i *_are almost completely bound (> 95%) to Mg^+2 ^at intracellular concentrations of Mg^+2^, while UDP is mostly bound (83%), and UMP, UDP-Glc, and Glc-1-P are only partially bound to Mg^+2 ^(~ 25%).

**Figure 4:**
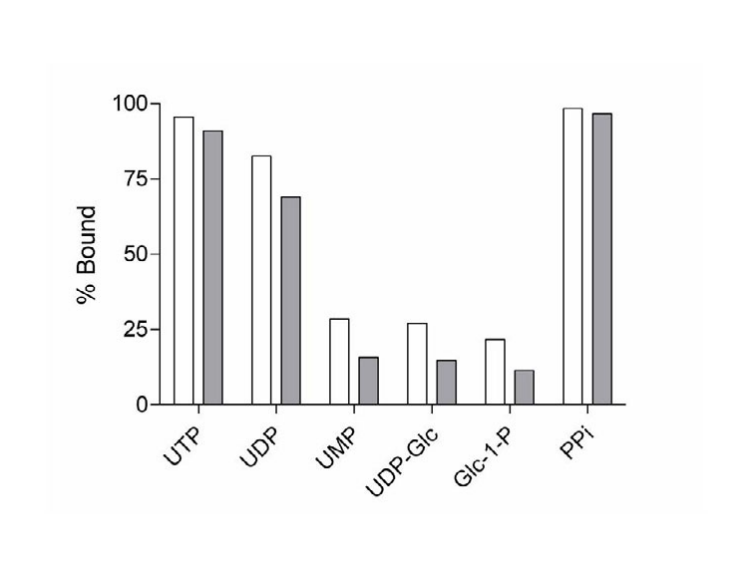
Percent of phosphate bound to Mg^+2 ^at a normal physiological Mg^+2 ^concentration of 1.5 mM (white) and at a hypomagnesaemia concentration of 0.7 mM (gray).

When the concentration of magnesium decreases, as is the case in hypomagnesaemia (0.7 mM Mg^+2^) there can be significant changes in the phosphate-magnesium concentration (Figure 4). In cases where Mg^+2 ^is tightly bound, PP_*i *_and UTP, there is not a large change in complex formation. However, when the binding is not as tight, as is the case with UDP, UMP, UDP-Glc, Glc-1-P, GlcNAc-1-P, and UDP-GlcNAc, the concentration of the phosphate-magnesium complex is greatly reduced by the change in magnesium ion concentration associated with hypomagnesaemia. This decrease in magnesium-phosphate complex formation would be expected to lead to decreased activity of glycogen synthase because of the role that AMP plays as an allosteric activator as well as decreasing the activity of phosphorylase *b *which is allosterically activated by glucose-6-phosphate.

To understand the role that metal binding plays in enzymatic activity we analyzed several known crystal structures of sugar NTs [39-49], GT-As [50-60] and GT-Bs [61-67]. The active site analysis of sugar NT crystal structures revealed the metal ion, Mg^+2^, ligands to be one to two oxygen atoms from the phosphates and two to three coordinated oxygens from aspartic acid residues from the enzyme in question [40,48]. Though not as complex, the coordination of Mg^+2 ^in sugar NTs is similar to the octahedral coordination site of Mn^+2 ^seen in GT-As, where the active site analysis shows two of the coordinating atoms to Mn^+2 ^are oxygens from the α- and β- phosphates and three to four coordination sites are direct interactions to the enzyme [50,52-54], [56-60]. A well-defined metal coordination site in GT-As allows for a high degree of coordination to occur between the metal, Mn^+2^, and the protein; this fact implies the presence of a permanent metal binding site [54]. This analysis coupled with the fact of a low intracellular concentration of Mn^+2 ^(10 nM) [38] makes the presence of a permanent metal binding site appropriate unless higher local metal concentrations can be maintained at the site of action of particular GTs. The enzymes that constitute GT-Bs, which are not metal dependent, have positively charged amino acid residues, four arginines, which are able to form hydrogen bonds directly to the phosphates [61-67].

## Conclusion

Our findings of no manganese-phosphate complex formation at physiological manganese ion concentration fits previously reported crystal structure determinations demonstrating the presence of a permanent binding site in GT-As for manganese [54]. For highest activity at low manganese concentration, the enzyme would need to have a way to permanently sequester manganese for full biocatalytic function. However for sugar NTs, which are magnesium dependent, the need for a permanent binding site is not required due to the much higher physiological concentration of divalent magnesium in healthy cells. As for inhibitors of sugar NTs, free UTP has been shown to be an inhibitor of Mg-UTP [35], and explains why UDP is an inhibitor in the presence of Mg^+2 ^while it was found not to bind to the enzyme in the absence of a divalent metal [35].

The role magnesium plays in the formation of metal-phosphate complexes may shed some light in why magnesium chloride therapy has lead to a decrease in serum glucose levels in patients with type 2 diabetes [20-23]. With the increase of the serum Mg^+2 ^concentrations, there will be an increase in the formation of magnesium-phosphate complexes. The complexes formed play a critical role by activating the biocatalysts involved in the manner in which glucose is biochemically stored and released. The decrease in biocatalyst function would not allow for the biochemical pathway to function properly, adequately sequestering glucose as glycogen when glucose levels are high and degrading glycogen when glucose levels are low. Obviously, these implications await human trials now that the basic biochemical data is available.

Knowledge of the method in which the metal binds to the substrate can also play an important role in drug design. When contemplating inhibitor design for sugar NTs, one must design the inhibitor such that it is able to compete against the most prevalent form of the substrate, which for this class of enzymes is the NTP-Mg^+2 ^complex. According to our results, the metal cation binds to NTP first and then after the reaction it leaves bound to PP_*i*_. In contrast, in designing inhibitors for glycosyltransferases, one needs to account for the binding that occurs between the manganese and the substrate because manganese is likely to be permanently bound in the active site and important in the catalytic activity of GT-As while not being important for the catalytic activity of GT-Bs. This knowledge of metal affinities therefore may aid in the development of drug candidates that are able to selectively inhibit one class of glycosyltransferases and phosphorylases over another and ultimately also may lead to diabetic drug development [25-28].

## Experimental

2-[4-(2-hydroxyethyl)-1-piperazinyl]-ethanesulfonic acid (Hepes), tetrasodium pyrophosphate decahydrate, magnesium chloride, and manganese (II) chloride were all purchased from Fischer Scientific Company (Hanover Park, IL). All other compounds were purchased from Sigma Chemical Co. (St. Louis, MO) unless otherwise noted and used without further purification. Nanopure water (18.1 MHz) prepared from a Barnstead E-pure water purification system was employed throughout.

### Isothermal titration calorimetry

ITC experiments were performed on a Microcal VP-ITC microcalorimeter (Northampton, MA) which was calibrated using the built-in electrical calibration check. All ITC experiments were conducted in 100 mM Hepes buffer pH 7.5 at 37°C. Nucleotide phosphate concentration was determined spectrophotometrically from UV absorbance measurements at 262 nm using an extinction coefficient of *ε*_262 _= 20,000 cm^-1 ^M^-1 ^[68]. Glucose-1-phosphate concentrations were determined via electrospray ionization mass spectrometry (ESI-MS) [69]. All solutions were degassed immediately prior to use. Titrant solution (2.5, 7.5, or 10 μL) was added from a 300 μL microsyringe at an interval of 200 sec. into the stirred sample cell (1.4288 mL) containing the nucleotide phosphate at a stirring rate of 480 rpm. To correct for heats of dilution, control experiments were performed by making identical injections of the titrant solution into a cell containing only buffer and these values were subtracted from the titration of the titrant solution into the reaction cell. The titrant solution contained the metal, Mg^+2 ^or Mn^+2 ^(75 mM), in Hepes buffer (100 mM, pH 7.5). The reaction cell contained 2.5 – 7.5 mM substrate (UTP, UDP, UMP, UDP-Glc, Glc-1-P, Glc, U, or PP_*i*_) in Hepes buffer (100 mM, pH 7.5). Data was analyzed using nonlinear least-squares curve fitting in Origin (7.0, OriginLab Corp., Northampton, MA) using the standard one binding site model supplied by Origin. This analysis yielded the thermodynamic parameters *K *(binding constant), enthalpy of binding (ΔH), entropy of binding (ΔS), and *n*, where *n *is the ratio of the metal ion to the substrate in the complex. See Additional file 1 for Isothermal titration calorimetry plots and tables containing *n*, *K*, ΔG, ΔH, and ΔS metal binding information for all substrates and metals and their thermograms.

## Abbreviations

ADP: Adenosine diphosphate; AMP: Adenosine monophosphate; ATP: Adenosine 5'-triphosphate; DNA: Deoxyribonucleic acid; DPA: Diphenylamine reagent; ESI-MS: Electrospray ionization mass spectrometry; G: Free energy of binding [cal/mol]; Glc: Glucose; Glc-1-P: Glucose-1-phosphate; GlcNAc: *N*-Acetylglucosamine; GlcNAc-1-P: *N*-Acetylglucosamine-1-phosphate; GT-A: Glycosyltransferase type A; GTB: Glycosyltransferase type B; H: Enthalpy of binding [cal/mol]; HDL: High-density lipoproteins; ITC: Isothermal titration calorimetry; K: Binding constant [M^-1^]; LDL: Low-density lipoproteins; n: Ratio of metal to substrate; NT: Nucleotidyltransferase; PP_*i*:_Pyrophosphate; S: Entropy of binding [cal/mol·T]; U: Uridine; UDP: Uridine diphosphate; UDP-Glc: UDP-glucose; UDP-GlcNAc: Uridine diphosphate-*N*-Acetylglucosamine; UMP: Uridine monophosphate; UTP: Uridine triphosphate; UV: Ultraviolet spectroscopy

## Authors' contributions

CJZ and GC-U collected ITC data, performed data analysis, and contributed to drafting the manuscript. NLP conceived of the study, participated in its design and coordination and contributed to drafting the manuscript. All authors read and approved the final manuscript.

## Supplementary Material

Additional file 1 Isothermal titration calorimetry plots and tables containing *n*, *K*, ΔG, ΔH, and ΔS metal binding information for all substrates and metals and their thermograms are given in additional file 1 (Table S1-S2, Figure S1-S22). Click here for file
