# Supplementary material for: Thermodynamics of binding of divalent magnesium and manganese to uridine phosphates: implications for diabetes-related hypomagnesaemia and carbohydrate biocatalysis
Source: Chem Cent J. 2008 Jul 15;2:15. doi: 10.1186/1752-153X-2-15 (PMC2490692; doi:10.1186/1752-153X-2-15)
Supplement: Additional file 1 — Isothermal titration calorimetry plots and tables containing n, K, ΔG, ΔH, and ΔS metal binding information for all substrates and metals and their thermograms are given in additional file 1 (Table S1-S2, Figure S1-S22). [file 1752-153X-2-15-S1.pdf]

SUPPORTING INFORMATION

Thermodynamics of Binding of Divalent Magnesium and Manganese to Uridine Phosphates: Implications for Carbohydrate Biocatalysis

Corbin J. Zea, Gulden Camci-Unal, and Nicola L. Pohl

Department of Chemistry and the Plant Sciences Institute, Iowa State University, Gilman Hall, Ames, IA, 50011-3111

E-mail: npohl@iastate.edu

Table of Contents

Table S1. Thermodynamic values for the binding of Mg^{+2} and Mn^{+2} to phosphates at 310 K S2

Table S2. Percentage of phosphates bound to Mg^{+2} and Mn^{+2} when the concentration of Mg^{+2} and Mn^{+2} is 1.5 mM and 10 nM respectfully at 310 K. S3

Figure S1- S11. Thermograms and binding isotherms of Mg^{+2} – phosphates S4-S9

Figure S12- S22. Thermograms and binding isotherms of Mn^{+2} – phosphates S9-S14

Table S1. Thermodynamic values for the binding of Mg^{+2} and Mn^{+2} to phosphates at 310 K.

Metal[a]	Substrate	*n*	*K* (M^{-1})	ΔG (cal/mol)	ΔH (cal/mol)	ΔS (cal/mol·T)
Mg^{+2}	UTP	0.991 ± 0.005	14,300 ± 700	-5878	3730 ± 30	31.0
Mg^{+2}	UDP	0.990 ± 0.004	3170 ± 70	-4952	3480 ± 20	27.2
Mg^{+2}	UMP	1.07 ± 0.07	270 ± 9	-3430	570 ± 10	12.9
Mg^{+2}	UDP-Glc	0.99 ± 0.05	250 ± 20	-3399	510 ± 40	12.6
Mg^{+2}	Glc-1-P	0.96 ± 0.02	185 ± 4	-3206	1040 ± 20	13.7
Mg^{+2}	PP*i*	1.030 ± 0.006	41,000 ± 2,000	-6539	-1770 ± 10	15.4
Mg^{+2}	UDP-GlcNAc	0.951 ± 0.100	110 ± 10	-2911	930 ± 200	12.4
Mg^{+2}	GlcNAc-1-P	0.775 ± 0.100	180 ± 20	-3180	1000 ± 100	13.5
Mg^{+2}	GlcNAc			No Binding		
Mg^{+2}	Glc			No Binding		
Mg^{+2}	U			No Binding		
Mn^{+2}	UTP	1.02 ± 0.02	13,000 ± 2000	-5829	2390 ± 60	26.5
Mn^{+2}	UDP	0.998 ± 0.008	2800 ± 2	-4885	2930 ± 30	25.2
Mn^{+2}	UMP	1.01 ± 0.03	300 ± 8	-3507	1640 ± 30	16.6
Mn^{+2}	UDP-Glc	1.00 ± 0.04	169 ± 8	-3165	1550 ± 80	15.2
Mn^{+2}	Glc-1-P	1.02 ± 0.01	340 ± 7	-3575	1760 ± 20	17.2
Mn^{+2}	PP*i*	1.02 ± 0.006	288,000 ± 2,200	-7747	-5614 ± 80	6.9
Mn^{+2}	UDP-GlcNAc	1.21 ± 0.03	124 ± 4	-2979	1550 ± 50	14.6
Mn^{+2}	GlcNAc-1-P	0.98 ± 0.02	179 ± 4	-3192	2510 ± 60	18.4
Mn^{+2}	GlcNAc			No Binding		
Mn^{+2}	Glc			No Binding		
Mn^{+2}	U			No Binding		

[a]Ratio of Metal to Substrate (*n*), binding constant (*K*), free energy (ΔG) as calculated from the free energy relationship ΔG = ΔH – TΔS, enthalpy of binding (ΔH), and entropy of binding (ΔS).

Table S2. Percentage of phosphates bound to Mg^{+2} and Mn^{+2} when the concentration of Mg^{+2} and Mn^{+2} is 1.5 mM and 10 nM respectfully at 310 K.

Metal	Substrate	% Bound[a]
Mg^{+2}	UTP	96
Mg^{+2}	UDP	83
Mg^{+2}	UMP	29
Mg^{+2}	UDP-Glc	27
Mg^{+2}	Glc-1-P	22
Mg^{+2}	PP*i*	98
Mg^{+2}	UDP-GlcNAc	14
Mg^{+2}	GlcNAc-1-P	21
Mn^{+2}	UTP	0
Mn^{+2}	UDP	0
Mn^{+2}	UMP	0
Mn^{+2}	UDP-Glc	0
Mn^{+2}	Glc-1-P	0
Mn^{+2}	PP*i*	0
Mn^{+2}	UDP-GlcNAc	0
Mn^{+2}	GlcNAc-1-P	0

[a]Percentage free uridine phosphate as determined form the binding constant, K.

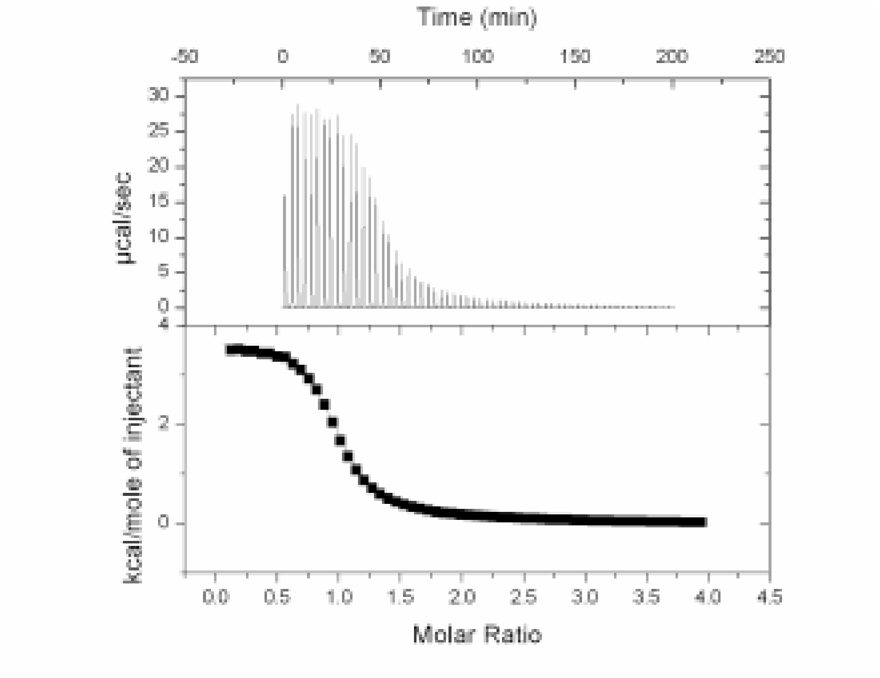

Figure S1. Thermogram (top) and binding isotherm (bottom) showing the addition of 75 mM $MgCl_2$ (syringe, 60 – 2.5 μL injections) into 2.1 mM UTP (cell) in 100 mM HEPES at pH 7.5 and 37 °C.

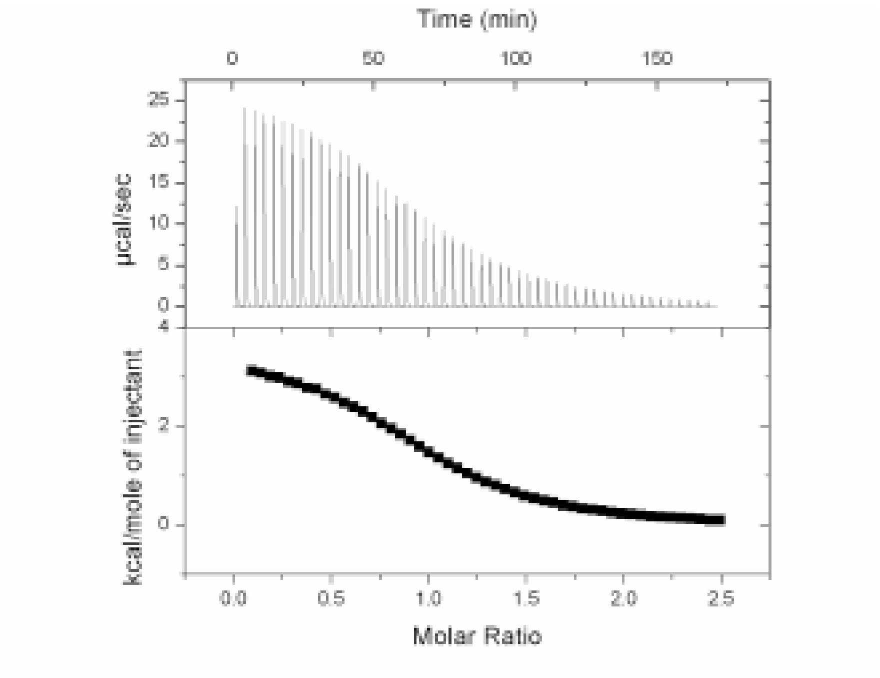

Figure S2. Thermogram (top) and binding isotherm (bottom) showing the addition of 75 mM $MgCl_2$ (syringe, 120 – 2.5 μL injections) into 2.8 mM UDP (cell) in 100 mM HEPES at pH 7.5 and 37 °C.

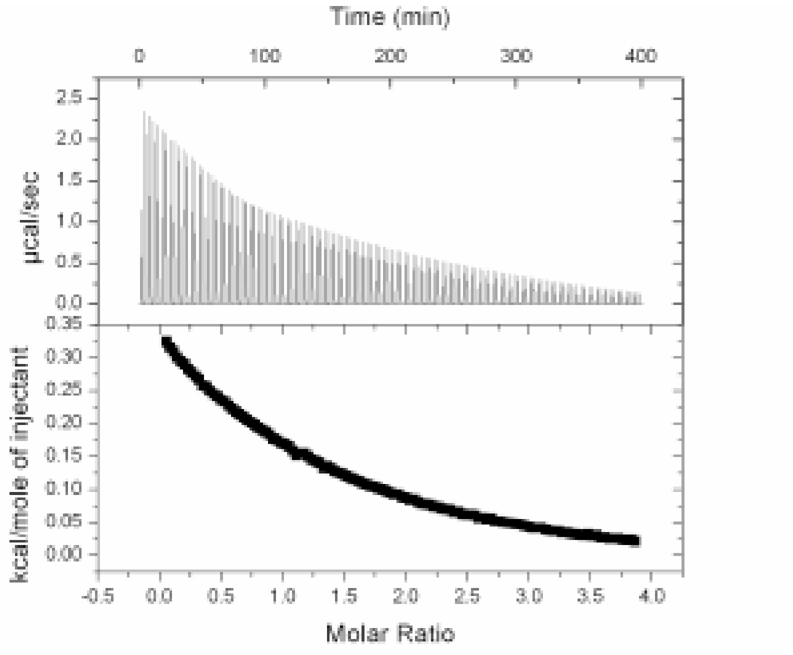

Figure S3. Thermogram (top) and binding isotherm (bottom) showing the addition of 75 mM $MgCl_2$ (syringe, 120 – 2.5 μL injections) into 4.5 mM UMP (cell) in 100 mM HEPES at pH 7.5 and 37 °C.

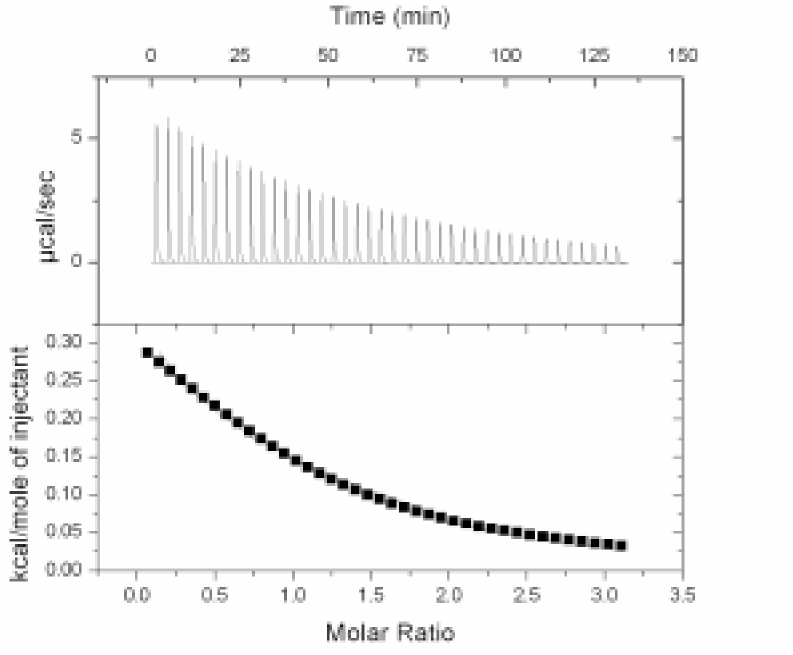

Figure S4. Thermogram (top) and binding isotherm (bottom) showing the addition of 75 mM $MgCl_2$ (syringe, 40 – 7.5 μL injections) into 5.6 mM UDP-Glc (cell) in 100 mM HEPES at pH 7.5 and 37 °C.

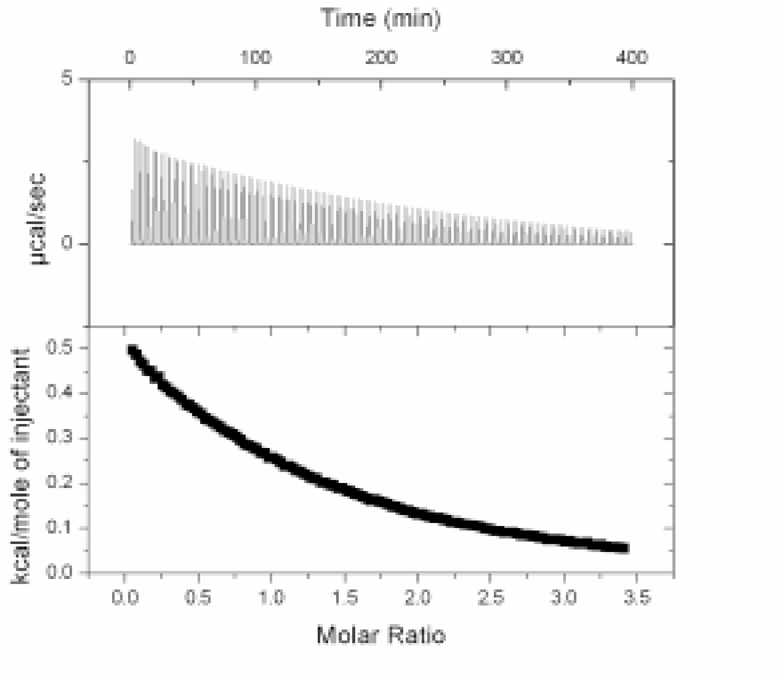

Figure S5. Thermogram (top) and binding isotherm (bottom) showing the addition of 75 mM $MgCl_2$ (syringe, 120 – 2.5 µL injections) into 5.1 mM α-D-glucose-1-phosphate (cell) in 100 mM HEPES at pH 7.5 and 37 °C.

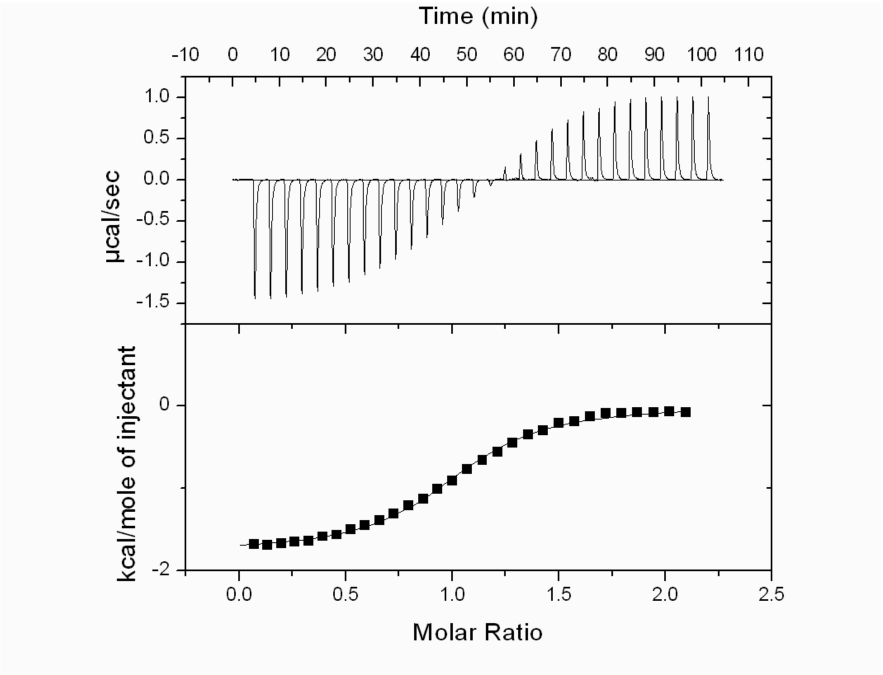

Figure S6. Thermogram (top) and binding isotherm (bottom) showing the addition of 4.5 mM $MgCl_2$ (syringe, 120 – 2.5 µL injections) into 0.5 mM pyrophosphate (cell) in 100 mM HEPES at pH 7.5 and 37 °C.

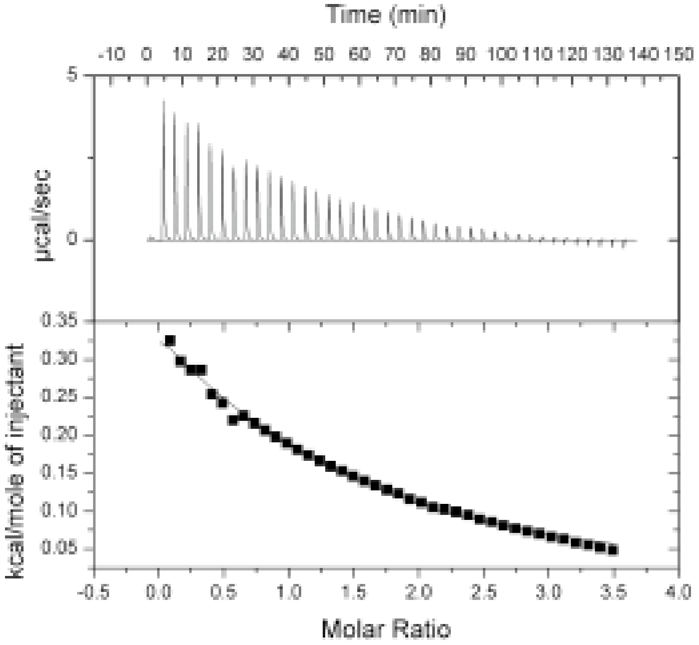

Figure S7. Thermogram (top) and binding isotherm (bottom) showing the addition of 75 mM $MgCl_2$ (syringe, 40 – 7.5 μL injections) into 5 mM UDP-GlcNAc (cell) in 100 mM HEPES at pH 7.5 and 37 °C.

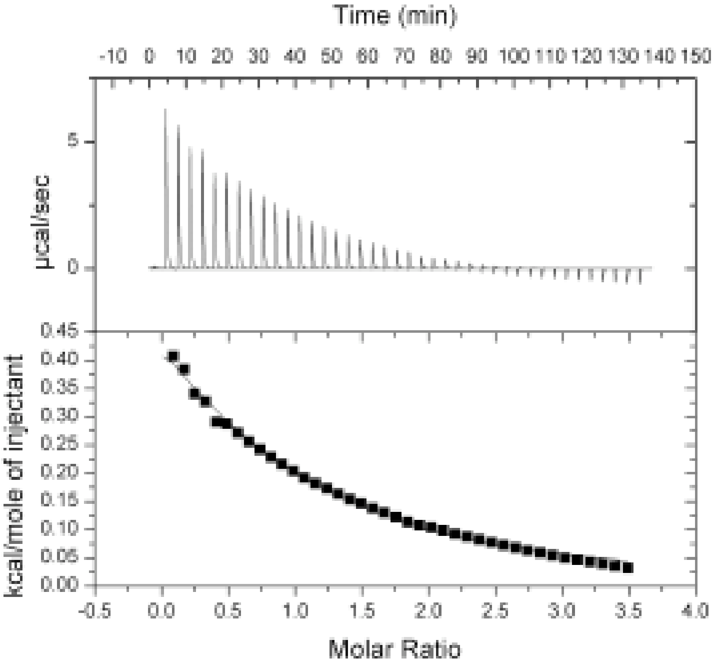

Figure S8. Thermogram (top) and binding isotherm (bottom) showing the addition of 75 mM $MgCl_2$ (syringe, 40 – 7.5 μL injections) into 5 mM GlcNAc-1-P (cell) in 100 mM HEPES at pH 7.5 and 37 °C.

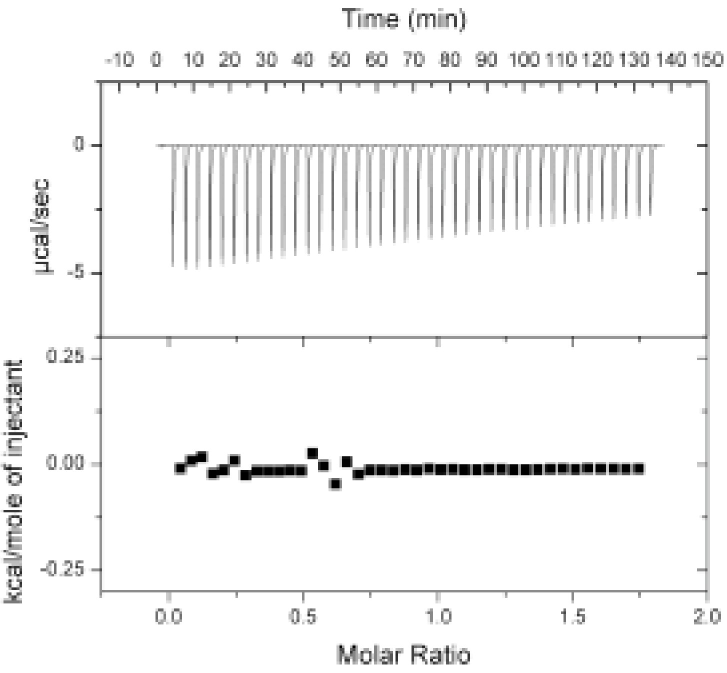

Figure S9. Thermogram (top) and binding isotherm (bottom) showing the addition of 75 mM $MgCl_2$ (syringe 40 _ 7.5 μL injections) into 10 mM GlcNAc (cell) in 100 mM HEPES at pH 7.5 and 37 ˚C.

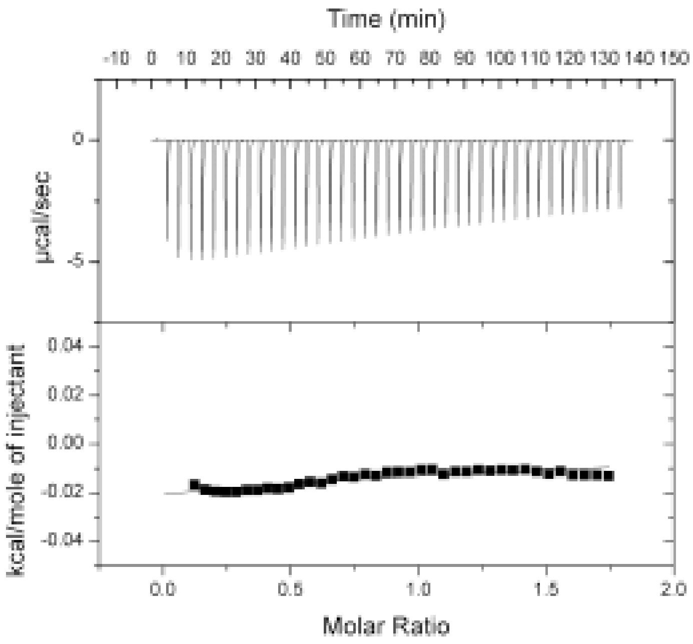

Figure S10. Thermogram (top) and binding isotherm (bottom) showing the addition of 75 mM $MgCl_2$ (syringe 40 _ 7.5 μL injections) into 10 mM glucose (cell) in 100 mM HEPES at pH 7.5 and 37 ˚C.

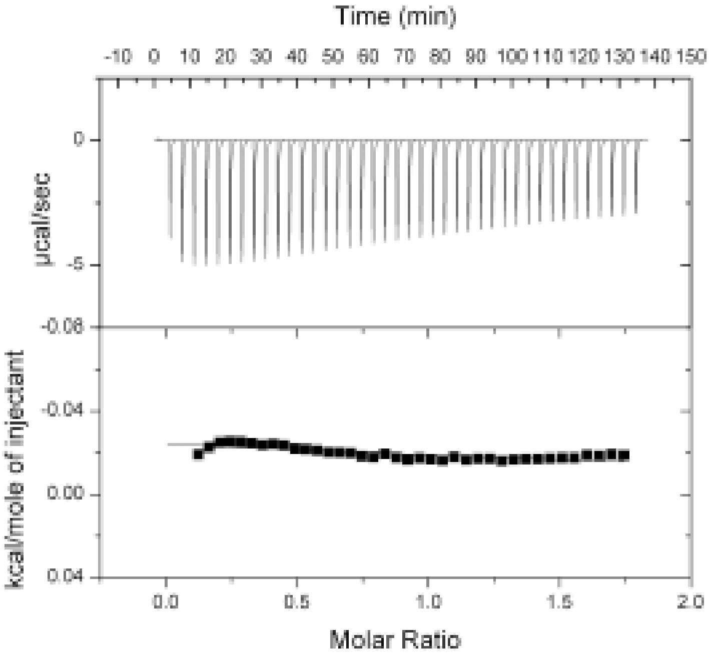

Figure S11. Thermogram (top) and binding isotherm (bottom) showing the addition of 75 mM $MgCl_2$ (syringe 40 _ 7.5 μL injections) into 10 mM uridine (cell) in 100 mM HEPES at pH 7.5 and 37 ˚C.

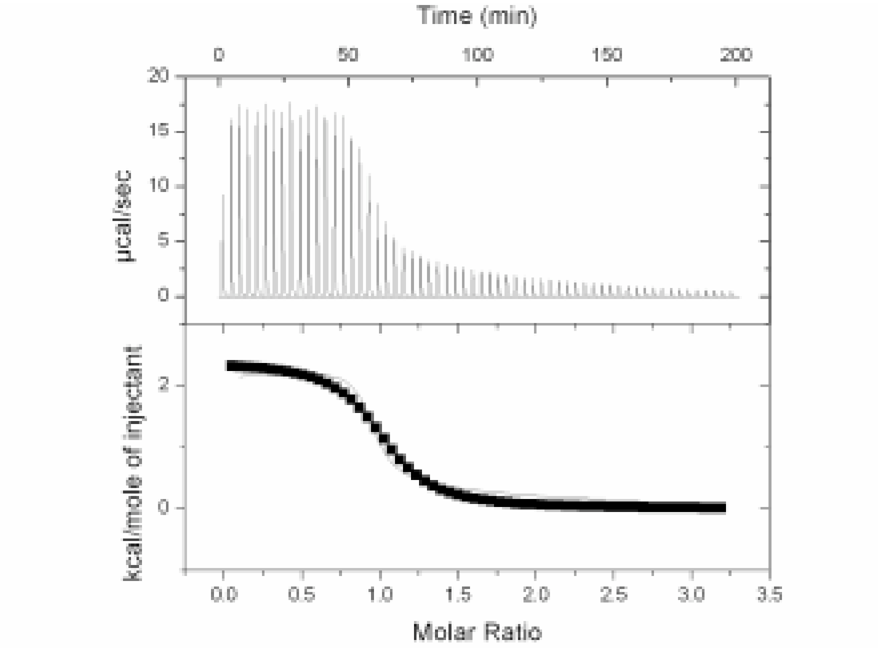

Figure S12. Thermogram (top) and binding isotherm (bottom) showing the addition of 75 mM $MnCl_2$ (syringe, 60 – 2.5 μL injections) into 2.5 mM UTP (cell) in 100 mM HEPES at pH 7.5 and 37 °C.

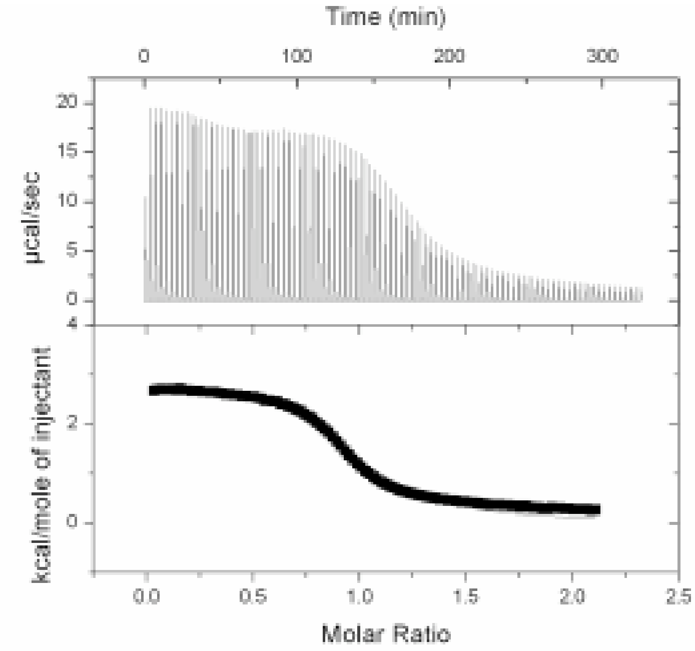

Figure S13. Thermogram (top) and binding isotherm (bottom) showing the addition of 75 mM $MnCl_2$ (syringe, 120 – 2.5 μL injections) into 7.5 mM UDP (cell) in 100 mM HEPES at pH 7.5 and 37 °C

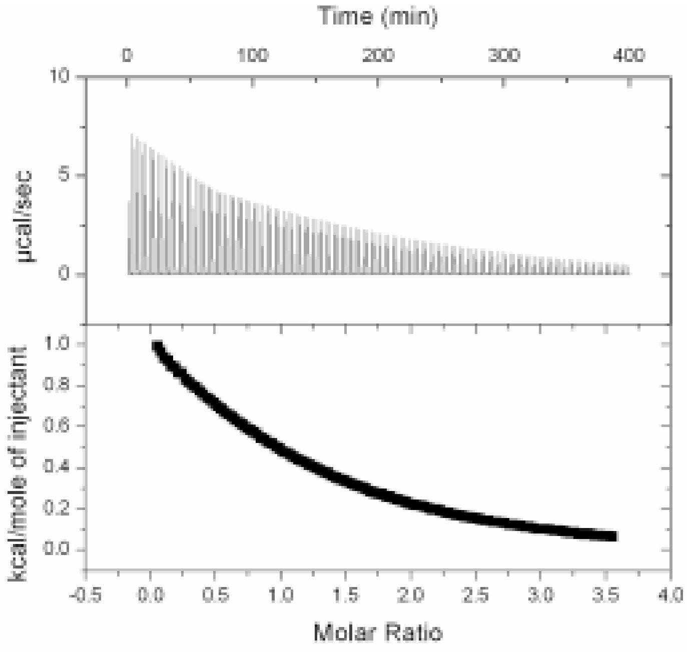

Figure S14. Thermogram (top) and binding isotherm (bottom) showing the addition of 75 mM $MnCl_2$ (syringe, 120 – 2.5 μL injections) into 4.9 mM UMP (cell) in 100 mM HEPES at pH 7.5 and 37 °C.

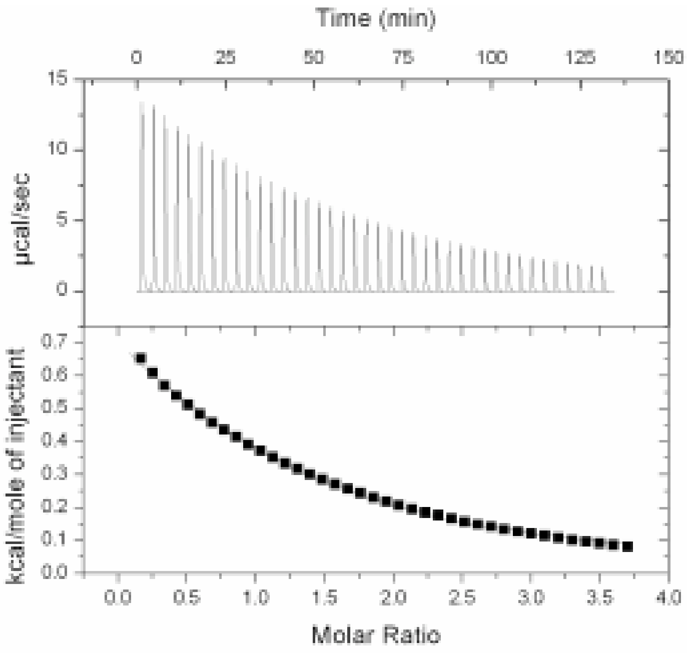

Figure S15. Thermogram (top) and binding isotherm (bottom) showing the addition of 75 mM $MnCl_2$ (syringe, 40 – 7.5 μL injections) into 4.7 mM UDP-Glc (cell) in 100 mM HEPES at pH 7.5 and 37 °C.

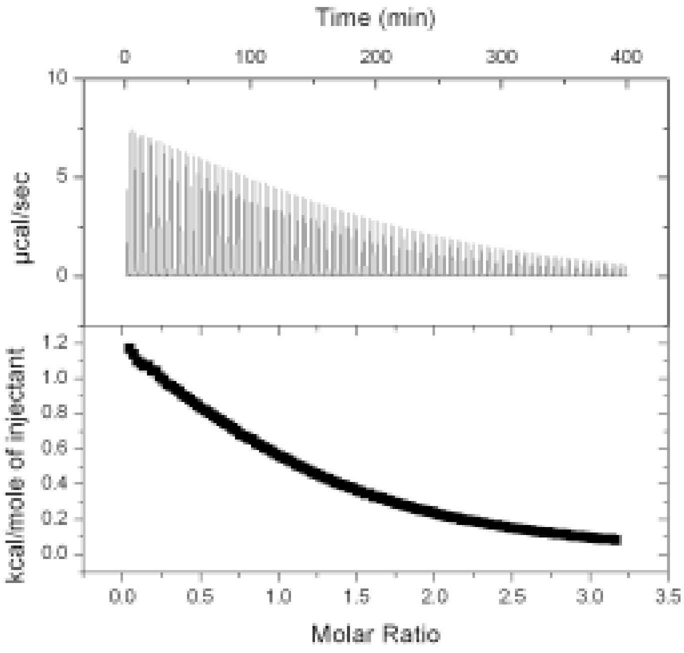

Figure S16. Thermogram (top) and binding isotherm (bottom) showing the addition of 75 mM $MnCl_2$ (syringe, 120 – 2.5 μL injections) into 5.5 mM α-D-glucose-1-phosphate (cell) in 100 mM HEPES at pH 7.5 and 37 °C.

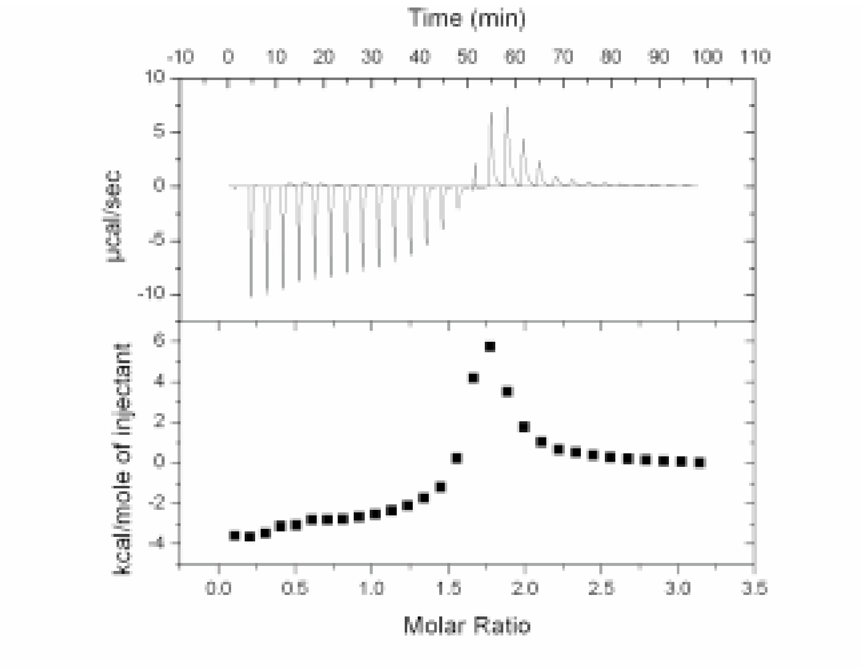

Figure S17. Thermogram (top) and binding isotherm (bottom) showing the addition of 4.5 mM $MnCl_2$ (syringe, 30 – 10 μL injections) into 0.5 mM pyrophosphate (cell) in 100 mM HEPES at pH 7.5 and 37 °C.

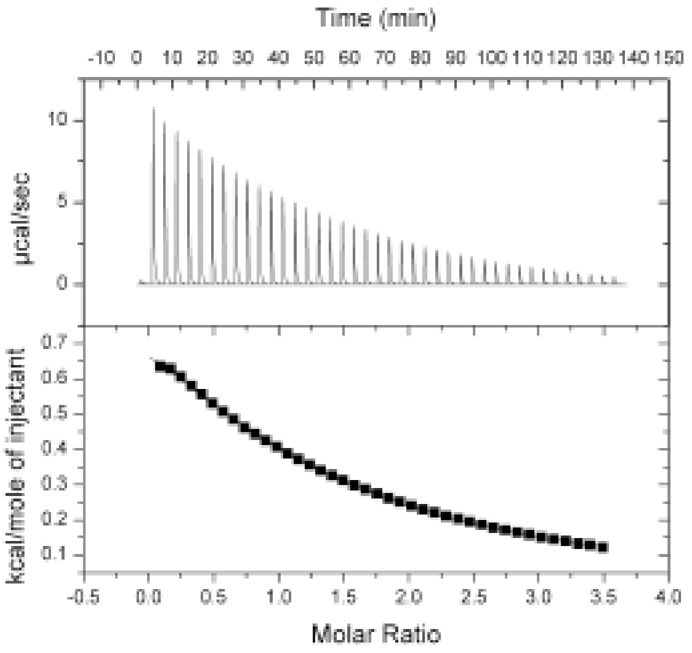

Figure S18. Thermogram (top) and binding isotherm (bottom) showing the addition of 75 mM $MnCl_2$ (syringe, 40 – 7.5 μL injections) into 5 mM UDP-GlcNAc (cell) in 100 mM HEPES at pH 7.5 and 37 °C.

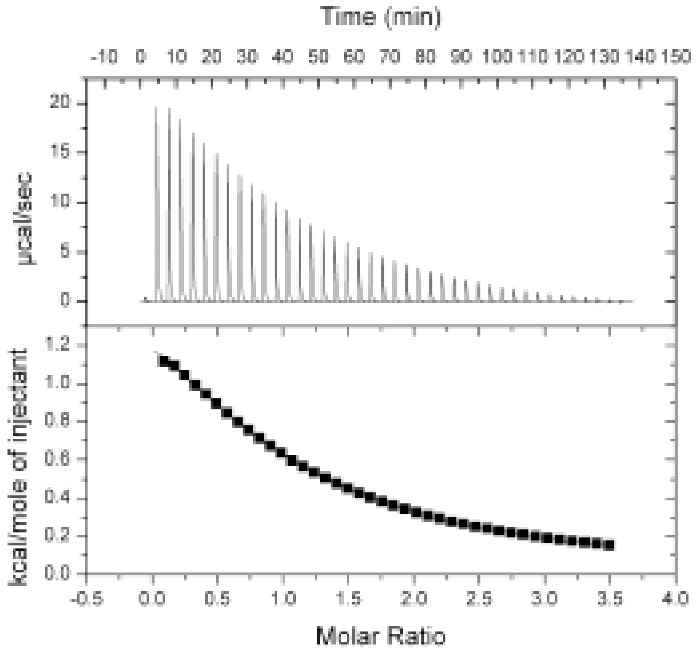

Figure S19. Thermogram (top) and binding isotherm (bottom) showing the addition of 75 mM $MnCl_2$ (syringe, 40 – 7.5 μL injections) into 5 mM GlcNAc-1-P (cell) in 100 mM HEPES at pH 7.5 and 37 °C.

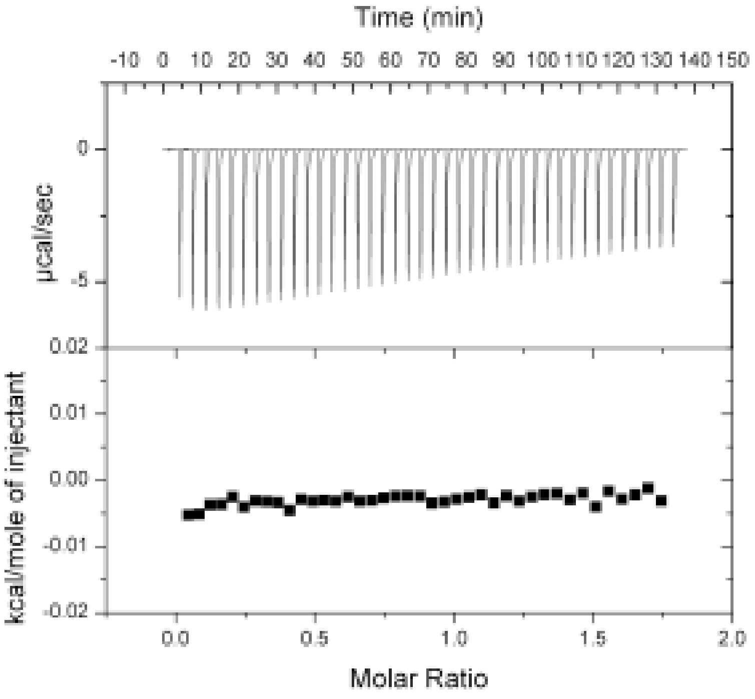

Figure S20. Thermogram (top) and binding isotherm (bottom) showing the addition of 75 mM $MnCl_2$ (syringe 40 _ 7.5 μL injections) into 10 mM GlcNAc (cell) in 100 mM HEPES at pH 7.5 and 37 ˚C.

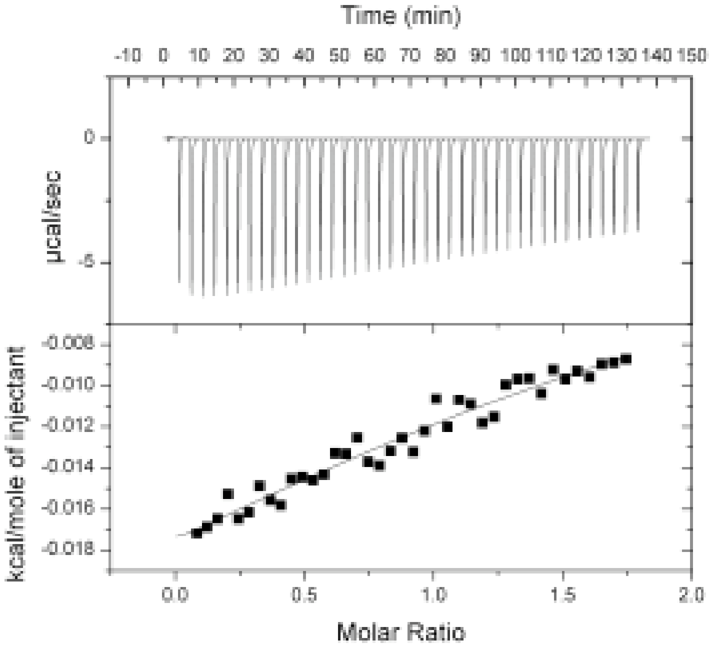

Figure S21. Thermogram (top) and binding isotherm (bottom) showing the addition of 75 mM $MnCl_2$ (syringe 40 – 7.5 µL injections) into 10 mM glucose (cell) in 100 mM HEPES at pH 7.5 and 37 ˚C.

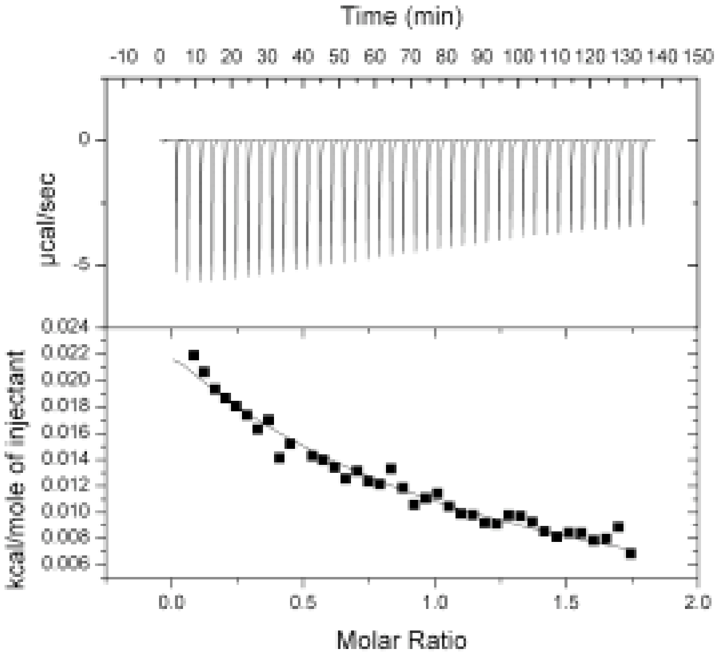

Figure S22. Thermogram (top) and binding isotherm (bottom) showing the addition of 75 mM $MnCl_2$ (syringe 40 – 7.5 µL injections) into 10 mM uridine (cell) in 100 mM HEPES at pH 7.5 and 37 ˚C.
